# Supplementary material for: REM sleep prefrontal high-frequency oscillation chains mediate distinct cortical – hippocampal reactivation patterns compared to NREM sleep
Source: eLife. 2026 Sep 7;15:RP110795. doi: 10.7554/eLife.110795 (PMC13549623; doi:10.7554/eLife.110795)
Supplement: Supplementary file 2 [file elife-110795-supp1.docx]

| **Animal** | **Epoch** | **NREM** | **REM** | **% REM** | **Animal** | **Epoch** | **NREM** | **REM** | **% REM** |
| --- | --- | --- | --- | --- | --- | --- | --- | --- | --- |
|  | 1 | 590 | 0 | 0.00% |  | 1 | 715 | 121 | 14.47% |
|  | 2 | 574 | 0 | 0.00% |  | 2 | 548 | 0 | 0.00% |
|  | 3 | 389 | 11 | 2.75% |  | 3 | 1064 | 13 | 1.21% |
|  | 4 | 362 | 0 | 0.00% |  | 4 | 1063 | 137 | 11.42% |
| **JS17** | 5 | 629 | 0 | 0.00% | **BG1** | 5 | 623 | 72 | 10.36% |
|  | 6 | 404 | 0 | 0.00% |  | 6 | 1778 | 275 | 13.40% |
|  | 7 | 1197 | 10 | 0.83% |  | 7 | 1636 | 263 | 13.85% |
|  | 8 | 1139 | 0 | 0.00% |  | 8 | 1284 | 270 | 17.37% |
|  | 9 | 916 | 74 | 7.47% |  | 9 | 693 | 27 | 3.75% |
|  | Total | **6200** | **95** |  |  | Total | **9404** | **1178** |  |
|  | 1 | 414 | 11 | 2.59% |  | 1 | 595 | 31 | 4.95% |
|  | 2 | 499 | 15 | 2.92% |  | 2 | 49 | 0 | 0.00% |
|  | 3 | 836 | 36 | 4.13% |  | 3 | 186 | 38 | 16.96% |
|  | 4 | 1124 | 0 | 0.00% |  | 4 | 678 | 0 | 0.00% |
| **JS13** | 5 | 1040 | 139 | 11.79% | **JS14** | 5 | 605 | 0 | 0.00% |
|  | 6 | 1926 | 120 | 5.87% |  | 6 | 901 | 167 | 15.64% |
|  | 7 | 1322 | 118 | 8.19% |  | 7 | 661 | 86 | 11.51% |
|  | 8 | 1440 | 122 | 7.81% |  | 8 | 594 | 126 | 17.50% |
|  | 9 | 1180 | 141 | 10.67% |  | 9 | 814 | 95 | 10.45% |
|  | Total | **9781** | **702** |  |  | Total | **5083** | **543** |  |
|  | 1 | 8 | 0 | 0.00% |  | 1 | 138 | 177 | 56.19% |
|  | 2 | 840 | 98 | 10.45% |  | 2 | 750 | 158 | 17.40% |
|  | 3 | 497 | 275 | 35.62% |  | 3 | 1066 | 124 | 10.42% |
|  | 4 | 1266 | 202 | 13.76% |  | 4 | 863 | 128 | 12.92% |
| **KL8** | 5 | 1571 | 113 | 6.71% | **JS21** | 5 | 435 | 14 | 3.12% |
|  | 6 | 629 | 182 | 22.44% |  | 6 | 649 | 0 | 0.00% |
|  | 7 | 1346 | 256 | 15.98% |  | 7 | 818 | 23 | 2.73% |
|  | 8 | 1193 | 243 | 16.92% |  | 8 | 859 | 13 | 1.49% |
|  | 9 | 568 | 41 | 6.73% |  | 9 | 743 | 0 | 0.00% |
|  | Total | **7918** | **1410** |  |  | Total | **6321** | **637** |  |
|  | 1 | 3 | 0 | 0.00% |  | 1 | 54 | 0 | 0.00% |
|  | 2 | 76 | 12 | 13.64% |  | 2 | 5 | 0 | 0.00% |
|  | 3 | 291 | 45 | 13.39% |  | 3 | 133 | 0 | 0.00% |
|  | 4 | 145 | 0 | 0.00% |  | 4 | 637 | 26 | 3.92% |
| **JS34** | 5 | 238 | 15 | 5.93% | **JS12** | 5 | 562 | 11 | 1.92% |
|  | 6 | 1513 | 274 | 15.33% |  | 6 | 1319 | 114 | 7.96% |
|  | 7 | 453 | 69 | 13.22% |  | 7 | 1178 | 83 | 6.58% |
|  | 8 | 535 | 75 | 12.30% |  | 8 | 1333 | 190 | 12.48% |
|  | 9 | 1070 | 112 | 9.48% |  | 9 | 720 | 0 | 0.00% |
|  | Total | **4324** | **602** |  |  | Total | **5941** | **424** |  |
|  | 1 | 731 | 30 | 3.94% |  | 1 | 25 | 0 | 0.00% |
|  | 2 | 435 | 0 | 0.00% |  | 2 | 504 | 16 | 3.08% |
|  | 3 | 742 | 0 | 0.00% |  | 3 | 885 | 34 | 3.70% |
|  | 4 | 865 | 0 | 0.00% |  | 4 | 919 | 14 | 1.50% |
| **JS15** | 5 | 1115 | 191 | 14.62% | **ZT2** | 5 | 1658 | 240 | 12.64% |
|  | 6 | 1476 | 0 | 0.00% |  | 6 | 1484 | 151 | 9.24% |
|  | 7 | 938 | 116 | 11.01% |  | 7 | 1573 | 47 | 2.90% |
|  | 8 | 1195 | 136 | 10.22% |  | 8 | 1804 | 239 | 11.70% |
|  | 9 | 990 | 224 | 18.45% |  | 9 | 716 | 12 | 1.65% |
|  | Total | **8487** | **697** |  |  | Total | **9568** | **753** |  |
