# Supplementary material for: REM sleep prefrontal high-frequency oscillation chains mediate distinct cortical – hippocampal reactivation patterns compared to NREM sleep
Source: eLife. 2026 Sep 7;15:RP110795. doi: 10.7554/eLife.110795 (PMC13549623; doi:10.7554/eLife.110795)
Supplement: Peer review history

# Editor report: eLife Assessment

**DOI:** 10.7554/eLife.110795.3.sa0

**Authors:** Caleb Kemere

**Affiliations:** Rice University United States

**Keywords:** Convincing, Important

Shin et al present significant new observations highlighting a novel oscillatory window during REM sleep that impacts neuronal dynamics and cross-regional communication and could have implications for our understanding of sleep's role in memory consolidation. This **important** study identifies and characterizes high-frequency oscillations (HFOs) in the prefrontal cortex (PFC) during REM sleep, reporting their temporal dynamics and coordination with hippocampal area CA1, and an intriguing dissociation between activation of CA1 neurons during REM HFOs and non-REM ripples that may impact sleep-dependent firing rate decreases. The main claims are supported with **convincing** evidence including an impressive range of analyses.

# Referee report: Reviewer #1 (Public review)

**DOI:** 10.7554/eLife.110795.3.sa1

Summary and Strengths:

Shin et al deepen our understanding of high frequency oscillations in the frontal cortex during REM in a manner that sheds important light on the roles of these events. In particular, they reveal that cortical HFOs are modulated by theta oscillations, occur in chains and recruit cortical neuronal activation patterns in a manner that is distinct from other high frequency events during nonREM or in hippocampus. They also show that these events occur during increased oscillatory cross-talk between hippocampus and cortex and may protect cortical neurons from down regulation of firing during sleep. Overall, this is important work with several novel observations pointing towards an important role for these events that will open become increasingly understood over time.

I also wanted to comment that 2D is a beautiful illustration of separate and essentially exclusive communication channels used during HF events in NREM vs REM. They almost perfectly complement each other's frequencies.

Weaknesses:

I have only one major scientific critique, I believe we need to see quantification of how phasic REM theta waves with versus without HFOs differ. What do REM HFOs add to the "normal" theta oscillation? Without this, comparison it is more difficult to interpret the meaning of these events. Given that HFO chains have IEIs around the time of a theta cycle duration, are the repeating spiking activities stronger during HFO repeats than during adjacent theta waves without HFOs? What percentage of theta waves contain HFOs and what is the firing rate during those theta waves with vs without HFOs? Is there differential firing rate modulation? The authors may even consider that all REM-HFO-specific quantifications should be shown as differential from phasic theta cycles without HFOs.

As a non-scientific comment on the manuscript itself: unfortunately, the paper is difficult to read and understand at times, requiring great effort by the reader. This is to an extent that communication is hindered. The paper is dense with changing methods often from panel to panel. Unfortunately, the panel quantifications are not explained in the results section in a manner that readers can understand without going to read the methods for often each individual panel. These measures should be explained in a way that lets readers understand the conclusions of each panel and grossly what calculations were used to reach those. Instead, too much jargon is used rather than clear descriptions of overall calculations being done for each panel.

The authors mention in discussion that they see increased functional connectivity between mPFC and CA1, but most data suggesting that seems to be based on LFP rather than spiking. Functional connectivity is defined best by spiking-spiking relationships. And these authors have spiking data. So I believe either the descriptive language should be pulled back to something like "oscillatory coupling" or more analyses should be dedicated to showing spike-spike coordination across regions.

Comments on revised version.

Previously raised concerns are addressed.

# Referee report: Reviewer #2 (Public review)

**DOI:** 10.7554/eLife.110795.3.sa2

Summary:

In this study, the authors investigate high-frequency oscillations (HFOs) in the prefrontal cortex during REM sleep. They identify a specific pattern where these HFOs occur in "chains" that are phase-locked to theta oscillations, primarily during the "phasic" periods of REM. The study contrasts these events with isolated HFOs and NREM ripples, suggesting a unique role for these chains in coordinating activity between the prefrontal cortex and the hippocampus. Most notably, the authors report that a specific subset of hippocampal cells-those that co-fire with the prefrontal cortex during these HFOs-increase their firing rates over the course of sleep, suggesting a potential mechanism for selective memory consolidation.

Strengths:

The study addresses an under-explored area of sleep physiology: the fine-grained temporal coordination between the cortex and hippocampus during REM sleep. The identification of HFO "chains" and their association with higher theta power provides an interesting framework for understanding how the brain might organize information transfer outside of NREM sleep. The observation that specific hippocampal populations show differential firing rate changes based on their participation in these HFO events is a striking finding that warrants further investigation.

Comments on revised version.

I do have one remaining concern, which is about their continued use of the term "reactivation" during REM sleep, whereas it still seems "activation" is more appropriate. The only place they show more Post vs. Pre activation is in Figure 6F/6G which includes NREM sleep where indeed reactivation is robust (but not the main focus of this paper). There is no evidence offered that the REM ensembles are not already "pre-configured" and active at similar levels (with similar activation patterns) during Pre sleep. Notably Louie and Wilson 2001 found greater "replay" during Pre than Post during REM. Also, the first half vs. second half comparisons (e.g. Fig 6C) could be more effectively performed in Figure 6A, showing that the same ordering persists across the periods. If this point were addressed, the significance of the findings could potentially increase.

# Referee report: Reviewer #3 (Public review)

**DOI:** 10.7554/eLife.110795.3.sa3

Summary:

Shin et al. examine hippocampal-prefrontal interactions during sleep using simultaneous CA1 and prefrontal cortex recordings in rats performing a spatial memory task. They identify high-frequency oscillation (HFO) events in PFC during REM sleep that occur in theta-modulated chains and are associated with increased CA1-PFC coherence and sequential, sparse reactivation of cortical ensembles. This pattern contrasts with the synchronous reactivation observed during NREM cortical ripples. Together with a simple cholinergic network model, the authors propose that REM HFO chains represent a distinct mechanism for hippocampal-cortical coordination that complements NREM ripple-mediated processing during sleep.

Strengths:

A major strength of the work is the extensive electrophysiological dataset, which includes simultaneous recordings of large neuronal populations in both hippocampus and prefrontal cortex across behaviour and subsequent sleep. The analyses linking high-frequency events to population dynamics, interregional coherence, and ensemble reactivation are technically sophisticated and provide an incredibly detailed description of REM-associated cortical activity patterns. In particular, the demonstration that REM HFOs occur in chains aligned to theta phase and organise sequential activation of cortical assemblies represents a potentially important advance in understanding the neural structure of REM sleep activity. The integration of experimental data with a computational model further provides a useful framework for interpreting the observed differences between REM and NREM network states in terms of neuromodulatory influences.

Weaknesses:

While overall this study provides a highly valuable body of work, there are two primary limitations, which if overcome, would provide substantially more significance to the overall characterisation of REM HFOs. Specifically:

Distinction from wake HFOs

The results largely support the authors' claim that REM HFO chains represent a distinct pattern of neural coordination compared to NREM cortical ripples. The analyses consistently show differences between REM and NREM events in terms of neuronal modulation, ensemble structure, and interregional coupling. However, similar high-frequency events during wake are not examined. Since REM sleep shares several network features with wakefulness, including strong theta oscillations, evaluating whether comparable PFC HFOs occur during wake would provide clarity on whether these events are specific to REM sleep (and its associated functions) or represent more general theta-associated phenomenon.

Link to memory consolidation

The manuscript proposes throughout that REM HFO chains may contribute to memory consolidation by coordinating hippocampal-cortical reactivation, but the evidence for this functional role remains indirect. The authors do highlight this as a limitation of the study - the inability to link their findings to learning - but it is not clear why. Further details of the behaviour results should be included. If no learning occurred across the eight behavioural sessions, this should be reported. If learning did occur, but could not be linked to HFO events, this should also be reported.

Comments on revised version.

The authors have since addressed these weaknesses. In supplementary figure S11 the authors now show that while HFOs were detectable during wake, they were not associated with gamma/theta oscillations or theta modulation of unit activity. This suggests that HFOs during REM are a distinct feature of REM sleep and not comparable to HFOs during NREM or wake. It would be interesting for future work to identify the significance of wake PFC HFOs, whether there are differences between HFOs during running compared to stationary behaviour, and their relationship to hippocampal sharp-wave ripples and memory consolidation.

Regarding the link between REM HFOs and memory consolidation, the authors have further acknowledged this as a limitation of the study and requirement for a more specific experimental design to test related hypotheses. Nevertheless, they do show a clear trajectory of learning in the rats and corresponding increase in reactivation of task-related activity which could be associated with REM sleep HFOs. This study paves the way for future experiments to more directly test this link.

# Author comment: Author response

**DOI:** 10.7554/eLife.110795.3.sa4

**Authors:** Justin D Shin, Michael Satchell, Paul Miller, Shantanu P Jadhav

**Affiliations:** Brandeis University Waltham United States; Brandeis University Waltham United States; Brandeis University Waltham United States; Brandeis University Waltham United States

The following is the authors’ response to the original reviews.

**Reviewer #1 (Public review):**

Summary and Strengths:

Shin et al deepen our understanding of high-frequency oscillations in the frontal cortex during REM in a manner that sheds important light on the roles of these events. In particular, they reveal that cortical HFOs are modulated by theta oscillations, occur in chains and recruit cortical neuronal activation patterns in a manner that is distinct from other high-frequency events during non-REM or in the hippocampus. They also show that these events occur during increased oscillatory cross-talk between hippocampus and cortex and may protect cortical neurons from downregulation of firing during sleep. Overall, this is important work with several novel observations pointing towards an important role for these events that will become increasingly understood over time.

I also wanted to comment that 2D is a beautiful illustration of separate and essentially exclusive communication channels used during HF events in NREM vs REM. They almost perfectly complement each other's frequencies.

Weaknesses:

I have only one major scientific critique: I believe we need to see quantification of how phasic REM theta waves with versus without HFOs differ. What do REM HFOs add to the "normal" theta oscillation? Without this comparison, it is more difficult to interpret the meaning of these events. Given that HFO chains have IEIs around the time of a theta cycle duration, are the repeating spiking activities stronger during HFO repeats than during adjacent theta waves without HFOs?

Here, we provide additional analyses to demonstrate that the phasic, theta-modulated PFC activity that we observe during HFOs is specifically tied to the occurrence of HFOs and not a strong phenomenon during non-HFO-associated theta periods. In Figure S5 M (middle and right), we find that aligning PFC multiunit activity to theta periods in phasic REM but temporally distant from HFOs does not elicit the same degree of theta-modulated activity as aligning to HFOs (as in Figure 2A and Figure S5 M, left).

Additionally, we provide analyses of the theta periods adjacent to HFOs (at different temporal thresholds) and demonstrate that this theta-modulated spiking activity is largely absent (Figure S5; compare to Figure 2A). Unlike Figure S5, this analysis was not restricted to putative phasic REM.

We have now added Supplementary Figures S5L-N to the revised manuscript.

What percentage of theta waves contain HFOs, and what is the firing rate during those theta waves with vs without HFOs? Is there differential firing rate modulation? The authors may even consider that all REM-HFO-specific quantifications should be shown as differential from phasic theta cycles without HFOs.

Although theta oscillations are continuously expressed during REM sleep, HFOs occur only intermittently, such that only a small subset of theta cycles contain HFOs. Across all animals and epochs included for analysis, we found that ~7.4% of theta cycles contain HFOs. We present an epoch-level quantification of this in Author response image 1, where the proportions were calculated across all, tonic, and phasic theta cycles. As expected, a higher proportion of putative phasic theta cycles contain HFOs.

Regarding differential firing rate modulation, we refer reviewer to normalized MUA plots in the manuscript (Figures 2A and 4F). We would like the emphasize that what we show is PFC multiunit activity that is normalized by the mean population firing rate during REM sleep. Thus, these figures, specifically the chain HFO aligned figure, indicate that there are peaks in activity around baseline level in the background of an overall decrease in population activity relative to baseline (i.e. the troughs surrounding the peaks have lower activity compared to baseline, as in Figure 9C). While this suggests that there is an overall decrease in firing rates during HFOs as compared to baseline theta periods without HFOs, this simply provides a qualitative account of this difference. In Figure S5N, we present firing rate comparisons during HFO chains versus theta periods during putative phasic REM bouts at least 4 theta cycles away from HFOs. We find that HFO chain-associated PFC neuron firing rates are lower compared to non-HFO theta periods, supporting our finding of activity suppression during HFOs.

**Author response image 1.** Proportion of theta cycles with HFOs. (A) Proportion of theta cycles with HFOs across all, tonic, and phasic cycles (***p = 4.90e-05, rank sum test).

Lastly, we appreciate the reviewer's suggestion that REM HFO quantifications could be framed relative to phasic theta cycles without HFOs. We agree that such comparisons are informative and ensure that the results we present are specific to periods with detected HFOs. In response, we have added additional analyses of theta periods outside of HFOs in Figure S5. Furthermore, while we found that a larger proportion of chain HFOs occurred during bouts of putative phasic REM compared to isolated events (Figure S5 and Figure S3C), most of the analyses that we performed were on events pooled across putative tonic and phasic, since putative phasic REM is relatively scarce (<10%).

We also refer the Reviewer to our response to Reviewer 2’s major comment #1 below where we reiterate several of our findings that demonstrate the HFO-specificity of the reported dynamics, as well as our extended response to Reviewer 3’s Public Review comment #1 where we show that the dynamics associated with REM HFOs are absent during HFOs detected during awake behavior (comparable theta state) on the W-Track (Figure S11). We hope that the additional control analyses we present as well as our expanded explanations adequately address the Reviewer’s concerns.

As a non-scientific comment on the manuscript itself: unfortunately, the paper is difficult to read and understand at times, requiring great effort by the reader. This is to an extent that communication is hindered. The paper is dense with changing methods, often from panel to panel. Unfortunately, the panel quantifications are not explained in the results section in a manner that readers can understand without going to read the methods, often for each individual panel. These measures should be explained in a way that lets readers understand the conclusions of each panel and what gross calculations were used to reach those. Instead, too much jargon is used rather than clear descriptions of the overall calculations being done for each panel.

We have now split and updated the figures in a more logical progression of ideas:

Figure 1: Prefrontal cortical HFOs in REM sleep using spectral analyses.

Figure 2: Characteristic spiking modulation in PFC during REM HFOs.

Figure 3: HFO and gamma distinction in theta cycles, and PFC-CA1 coherence (including chain/ isolated HFOs in phasic and tonic REM stages).

Figure 4: Differential modulation of PFC spiking activity during REM PFC HFOs vs. NREM PFC ripples.

Figure 5: Characteristic PFC population activity during REM HFO chains.

Figure 6: Comparison of PFC reactivation during REM PFC HFOs vs. NREM PFC ripples.

Figure 7: Differential engagement and excitability modulation of CA1 neurons by REM HFOs.

Figure 8: REM theta phase shifting CA1 neurons preferentially engaged by REM HFOs.

Figure 9: (Model) Network model with ACh reproduces spiking modulation during REM PFC HFOs vs NREM PFC ripples.

Figure 10: (Model) Model reproduces restricted REM coactivity vs. widespread NREM coactivity.

We have also rearranged the figures to parallel the main figures and results section.

The authors mention in the discussion section that they see increased functional connectivity between mPFC and CA1, but most data suggesting this seems to be based on LFP rather than spiking. Functional connectivity is best defined by spiking-spiking relationships. And these authors have spiking data. So I believe either the descriptive language should be pulled back to something like "oscillatory coupling" or more analyses should be dedicated to showing spike-spike coordination across regions.

We have updated the manuscript accordingly. Specifically, we have modified the text on Page 13, Lines 23-24:

“These chains are associated with increased measures of oscillatory coupling between PFC and CA1…”

**Reviewer #1 (Recommendations for the authors):**

(1) Please ensure that analytical methods are presented in the same order in the methods section as they are in the results section - panel by panel. That said, the methods section is well-written and presented.

We apologize for any confusion this may have caused. We have now reorganized the methods section to ensure they presented in the same order as the panels in the main figures.

(2) Please specify whether the recordings/behaviors occur during the animal's light circadian phase.

We have added a statement in the methods under the “Behavior” section on Page 34, Lines 9-12 indicating that the experiments took place during the light phase:

“During the recording day, animals were introduced to the novel W-maze (~80 × 80 cm with ~7 cm wide tracks) for the first time and learned the task rules over eight behavioral sessions during the animals’ light phase between the hours of 9 AM and 6PM.”

(3) Please specify how many tetrodes are in mPFC and how many in CA1?

We have added this clarification on Page 33, Lines 27-28:

“Tetrodes were split equally between PFC and CA1 (15, 16, or 32 tetrodes in each region).”

(4) All mentions of "coherence" should have frequency bands specified. "Theta coherence", for example.

We have now added this information to all relevant mentions of “coherence”.

(5) The intuitive logic of the phase slope index (2K) should be briefly explained for maybe half a sentence in the results section. The intuition should be explained better in the methods section devoted to it.

We have added more clarification on the phase slope index method, both in the legend of Figure 3 on Page 21, Lines 22-23:

“Phase slope index (PSI), which is a measure of phase lag consistency across different frequencies…” and in the methods section under “Phase slope index” on Page 39, Lines 21-26:

“In practice, PSI is used to assess the consistency of phase lag relationships between two signals across different frequency bands and is a measure that is weighted by oscillatory coherence. We opted to use PSI to estimate the directional flow of information instead of other methods, such as Granger causality, since it has been demonstrated that PSI is less prone to false positives.”

(6) "Cofiring" and "coactivity" should be defined clearly as measures - preferably in the results section if possible. They sound similar and are somewhat jargon-y without self-explanatory meaning (or difference from each other). How should readers understand and interpret them?

We apologize for the confusion regarding these two terms, which are both used throughout the manuscript. Here, we specifically used to term “cofiring” to specify the explicit quantification of coincident activity between pairs of neurons (e.g. Figure 4E, left) as described in the Methods section under “Ripple and HFO co-firing”. There were instances where “coactivity” was used to refer to this quantification, and they have been changed to “cofiring”. We have now added a statement to the manuscript to clarify that “cofiring” is a quantification of coincident activity between neurons on Page 7, Lines 34-35:

“Overall cortical co-firing, which is a measure of coincident activity between neuron pairs during discrete events”

Additionally, the term “coactivity” in the manuscript is used when describing neuronal activity in the model or when there are mentions of coincident activity other than the explicit quantification described above.

(7) The temporal threshold for "cofiring" should be stated in the results to enable interpretation of the results.

We apologize for the lack of clarity regarding the cofiring metric. Here, the temporal threshold that we are imposing is determined by the ripple/HFO event times (see Methods section “LFP event detection”). If both neurons in a pair emit spikes within the defined window of an event, they are considered to have “cofired” (Cheng and Frank 2008, Singer and Frank 2009, Sosa, Joo et al. 2020).

We have now clarified this in the results section on Page 7, Lines 34-35:

“Overall cortical cofiring, which is a measure of coincident activity between neuron pairs during discreet events…”

We have also added a clarifying statement in the Methods section under “Ripple and HFO cofiring” on Page 40, Lines 24-25:

“Here, cofiring assesses coincident activity between neuron pairs within the start and end times of events, and thus, no explicit temporal threshold was implemented.”

(8) Please clarify more systematically in which region the NREM ripples were detected. The natural assumption is the hippocampus, but at times it is mentioned that they are detected in the cortex. Are they cortical in all analyses? Readers could easily get confused about this and misinterpret "ripples". To clarify further, if these are always cortical events, I suggest renaming "ripples" in this text to "cortical NREM HFOs".

We apologize for the confusion. For all mentions of “ripples” in the text, we are referring to PFC ripples specifically in NREM sleep. When hippocampal ripples are mentioned, we differentiate them by explicitly using “sharp-wave ripples” or “SWRs”. Our decision to use “ripples” for NREM sleep was based on previous studies that investigated NREM high-frequency events in cortex (Khodagholy, Gelinas et al. 2017, Helfrich, Lendner et al. 2019, Vaz, Inati et al. 2019, Aleman-Zapata, Morris et al. 2022, Ghosh, Yang et al. 2022, Shin and Jadhav 2024). Furthermore, we elected to use the “HFO” nomenclature for high-frequency events in REM sleep, since it has been used in previous studies to describe these events (Tort, Scheffer-Teixeira et al. 2013, Bueno-Junior, Ruckstuhl et al. 2023). Thus, to remain consistent with the literature, we decided to use these terms to describe these sleep-state-specific events throughout the manuscript:

Hippocampal sharp-wave ripples (SWRs) in NREM sleep

PFC ripples in NREM sleep

PFC HFOs in REM sleep

We have now added the following statement on Page 5, Lines 1-3:

“However, to avoid ambiguity, and to conform to previous nomenclature, we refer to cortical NREM events as ripples, cortical REM events as HFOs, and hippocampal sharp-wave ripples in NREM as SWRs throughout.”

(9) The analysis performed for 4B is not explained clearly. It is somewhat better explained in the methods. I believe the reader should understand that each HFO is treated as an event, and spiking participation per unit was measured, and then the similarity of that pattern was assessed between HFOs. I also find the x-axis being quartiles makes understanding this graph particularly difficult.

Why not label by raw lag and show a correlation plot rather than break down by quartiles? Alternatively, labeling the millisecond values of these quartiles on the x-axis labels may make comprehension much easier.

We apologize for the lack of clarity regarding this method. We have added points to clarify the analytical procedure used for Figure 4B (Now Figure 5B) on Page 8, Lines 25-28:

“We represented each HFO as a binary vector of PFC neurons active during the event, and computed the Pearson correlation between the vectors of every pair of consecutive HFOs” As well as in the Figure 5 legend on Page 24, Lines 7-10:

“Here, the PFC spiking activity during each HFO was binarized across all neurons and the Pearson correlation coefficient was calculated between adjacent events as a measure of pattern similarity. Then the relationship between pattern similarity and IEI was reported.”

In addition to the quartile labels, we have now added the average inter-event interval (IEI) of each quartile to the x-axis labels of Figure 5B to improve comprehension as well as a statement in the figure legend on Page 24, Line 11:

“Below each quartile is the average IEI of that quartile in milliseconds.”

(10) "Rank order analysis" should be again defined in the results in a manner that the reader can follow the point of the analysis and figure. For example, the goal is to assess the spike sequence across HFO events by looking at the regularity of spike timing rank for each neuron in each HFO event. Also, could this correlation be more simply calculated and presented as just a standard deviation around the mean of that cell's rank?

We apologize for not including an explanation of this analysis in the results section. We have now added more detail about the rank order analysis to the Figure 5 legend on Page 24, Lines 18-21:

“The mean rank-order correlation from the leave-one-out cross-validation procedure. Each event’s rank was correlated with the averaged rank across all other events. The average across all events compared to a distribution of means generated by jittering (n = 1000) spike times is shown.”

We also provided a short description of the procedure in the results section on Page 8, Lines 32-36:

“Furthermore, we examined whether the order in which individual PFC neurons fired during HFOs within a chain was preserved across chains. For each chain, we extracted each cell's first-spike rank order and compared it to a leave-one-out template constructed from the average normalized rank across all other chains.”

Regarding the Reviewer’s second comment about the correlation, if we presented the result as the standard deviation around the mean of the cell’s rank, the result would be similar to the example rank order in Figure 5C, which we provided as a visualization of the rank-order template procedure we used. However, this would not necessarily demonstrate the consistency of population activity across HFO chains. To demonstrate consistency in activity across HFO chains, we used a leave-one-out cross-validated approach where each chain event was assessed separately. For each event, the neurons firing during that event was ranked and normalized 0-1. Then, the average rank of each neuron across all other events was calculated. Lastly, the correlation between the ranks of the left-out event and the average ranks of the template was taken to assess similarity in sequential activity. We opted to use this method since it has been demonstrated to be effective for evaluating similarities in sequential across events (Stark, Roux et al. 2015, Valero, Viney et al. 2021).

(11) In Figure 5B and the related results section, I gather that "spatial" relates to place field location rather than anatomical/tetrode location of the neuron? This was not my original understanding and should be stated clearly.

We apologize for the lack of clarity regarding this result. Yes, the term “spatial” refers to spatial rate map correlation between CA1 neuron pairs, specifically during the behavioral W-Track session prior to the sleep session where cofiring was assessed. We have updated the y-axis label of Figure 5B (Now Figure 7B) to include “rate map” and have updated the results section on Page 9, Lines 32-33:

“…we observed a higher degree of spatial rate map correlation for high cofiring pairs…”

(12) Figure 5E and its caption are almost totally unable to be explained since axes aren't explained well in either. It is only by inference from the results text that meaning can be assumed.

We apologize for the lack of clarity regarding Figure 5E (Now Figure 7E). We have now updated the y-axis labels on both updated figures (Figures 7E and 8B) and have reworded and added more detail to the figure legend on Page 28, Lines 1-5:

“High REM PFC HFO cofiring CA1 neurons exhibited a greater degree of suppression during NREM PFC ripples. For a description of modulation index, see Methods section Ripple/HFO aligned modulation. Here, since CA1 neurons exhibit a robust decrease in activity in response to NREM PFC ripples, we refer to the modulation as suppressive.”

(13) Figure 5F is interesting, supporting the concept that HFOs "protect" neurons from downscaling. However, how can a "neuron" be cofiring? Would cofiring not be defined in a pairwise manner, and so each unit of the cofiring measure would be a pair of neurons? This question applies to other panels in this figure. Please clarify this.

We apologize for the lack of clarity regarding the exact cofiring metric that we use. For Figures 7D-F and 8A, since we wanted to relate cofiring to changes in firing rate and modulation state during NREM PFC ripples, we calculated single cofiring values for each CA1 neurons by averaging across all pairings with PFC neurons. Thus, this metric gives us an estimate of the overall cofiring strength of each CA1 neuron. We have now added a new section in the Methods under “Calculation of a single cofiring metric and separation into populations of high and low cofiring neurons” on Page 44, Lines 34-43:

“Since we wanted to relate the above cofiring metric to other measures, we needed to obtain a single-value cofiring metric for each neuron. To do this, we averaged the cofiring values across all pairings for a neuron (e.g. 1 CA1 neuron paired with all PFC neurons) and reported it as the cell’s cofiring. Furthermore, since we observed a bimodal distribution of CA1-PFC cofiring values in REM sleep, we split the population based on whether the average (across all cell-cell combinations) cofiring value or correlation coefficient of a cell was above or below 0 (High cofiring > 0; low cofiring < 0). Also, since the NREM ripple cofiring distribution was unimodal, we additionally split the populations using the mean of the average cofiring or correlation coefficient distributions (High cofiring > mean; low cofiring < mean).”

We have also clarified this in the Figure 7 legend on Page 27, Lines 25-28:

“For comparisons between cofiring and other metrics (e.g. firing rate), a single cofiring value was calculated for each neuron by averaging the cofiring metric across all neuron pairings. Additionally, high and low cofiring CA1 neurons were split based on average cofiring values > 0 and < 0, respectively.”

**Reviewer #2 (Public review):**

Summary:

In this study, the authors investigate high-frequency oscillations (HFOs) in the prefrontal cortex during REM sleep. They identify a specific pattern where these HFOs occur in "chains" that are phase-locked to theta oscillations, primarily during the "phasic" periods of REM. The study contrasts these events with isolated HFOs and NREM ripples, suggesting a unique role for these chains in coordinating activity between the prefrontal cortex and the hippocampus. Most notably, the authors report that a specific subset of hippocampal cells-those that co-fire with the prefrontal cortex during these HFOs-increase their firing rates over the course of sleep, suggesting a potential mechanism for selective memory consolidation.

Strengths:

The study addresses an under-explored area of sleep physiology: the fine-grained temporal coordination between the cortex and hippocampus during REM sleep. The identification of HFO "chains" and their association with higher theta power provides an interesting framework for understanding how the brain might organize information transfer outside of NREM sleep. The observation that specific hippocampal populations show differential firing rate changes based on their participation in these HFO events is a striking finding that warrants further investigation.

Weaknesses:

The primary weakness of the study lies in the lack of a clear distinction between global brain states and the specific events being analyzed. Because the authors compare HFOs across different sleep stages (NREM, tonic REM, and phasic REM) without sufficient controls, it is difficult to determine if the observed differences are intrinsic to the HFOs themselves or simply a reflection of the different physiological states in which they occur.

We would first like to note in case it was unclear – as clearly noted in our manuscript title, state-dependence is an integral part of our results, with the primary comparison in the manuscript being between REM cortical HFOs and NREM cortical ripples, and correspondingly spiking activity patterns observed in prefrontal cortical-hippocampal circuits during these event-types that occur in the two sleep stages. As noted in response to Reviewer 1’s Comment #8 and Reviewer 3’s Comment #1, to avoid ambiguity and to remain consistent with existing literature, we use the following terms to describe these sleep-state specific events throughout the manuscript: Hippocampal sharp-wave ripples (SWRs) in NREM sleep, PFC ripples in NREM sleep, and PFC HFOs in REM sleep.

We refer the Reviewer to our Response to Reviewer 1’s first comment where we provide additional analyses of theta periods outside of HFO times (i.e. baseline REM periods), including Figure S5. We also further address these comments as a response to Reviewer 2’s major comment #1 below.

Furthermore, the evidence for "structured reactivation" is not yet convincing. The temporal alignment of these reactivation events appears inconsistent, with peaks occurring well before the HFO itself, and the analysis does not sufficiently control for pre-existing cellular assembly strengths.

We have now addressed this as a response to Reviewer 2’s major comments #3 and #8 below, including Figure 6.

Additionally, some of the sleep architecture presented appears atypical, such as very short REM bouts and direct NREM-to-REM transitions that bypass standard progression, raising questions about the consistency of the sleep detection across animals.

We have now addressed this as a response to Reviewer 2’s major comment #2 below, including Figure S1 and Figure S3. We expect that addition of these figures will mitigate concerns about our sleep staging procedures and will clarify certain points raised by the Reviewer.

Finally, the study does not account for potential confounds like baseline firing rates when interpreting the behavior of "high-cofiring" neurons, which may simply be the most active cells in the population.

We have now addressed this as a response to Reviewer 2’s major comment #6 below.

**Reviewer #2 (Recommendations for the authors):**

In this study, the authors detect activity periods during REM sleep that feature high-frequency oscillations in the prefrontal cortex. They term these REM HFOs and report that they occur in "chains" phase-locked to theta oscillations. They contrast these with HFOs that appear in isolation and with ripples observed during NREM sleep, either in the PFC or in the hippocampus. The data presented is very interesting in places. The authors show that these HFOs are observed primarily during "phasic REM" periods that have higher theta power. It appears that overall firing is substantially lower surrounding these events. Intriguingly, it appears that CA1 cells that co-fire with the PFC during HFOs increase their firing rates over the course of sleep, whereas other neurons show a firing decrease. This seems to be the most striking finding of this study.

Major:

(1) The findings are generally intriguing, but the study makes some choices that are hard to understand. They begin comparing HFOs that occur in chains to HFOs that occur in isolation, even though it appears that chained and isolated HFOs likely occur at different times, some during tonic REM, some during phasic REM, and others during NREM. The lack of control for sleep state makes it difficult to determine if the reported differences are intrinsic to the HFO patterns or merely reflections of the underlying global brain state. Overall, I just didn't quite understand the motivation for comparing isolated and chain HFOs, as it seems natural that chains would occur during periods of greater synchrony.

We appreciate the Reviewer for raising these points and agree that the properties of ripples and HFOs cannot be interpreted independently of the underlying brain state. As we mentioned in the initial response, we do expect that the generation of these ripples/HFOs in NREM and REM sleep are inextricably linked to global brain state (ex., cholinergic tone, as shown in the model in Figures 9-10), which results in differing patterns of activity across sleep states. We noted in the response to the public review comment #1 above that as clearly noted in our manuscript title, state-dependence is an integral part of our results, with the primary comparison in the manuscript being between REM cortical HFOs and NREM cortical ripples, and correspondingly, spiking activity patterns observed in cortical-hippocampal circuits during these event types that occur in the two sleep stages.

Sleep state: Our primary goal in comparing isolated and chain HFOs (chain vs. isolated HFOs are only compared in REM periods, not NREM periods) was not to suggest that the differences that we observe are state-independent, but rather to provide evidence that temporal clustering of HFOs underlies distinct PFC dynamics, as well as enhanced CA1 engagement. Rather than attempting to dissociate ripple/HFO occurrence and the differential physiology that underlie NREM and REM sleep states, we view them as complementary—both sleep states are permissive to generation of high-frequency oscillations. Similarly, putative tonic and phasic REM substates (low vs high theta power) are differentially permissive for isolated and chain HFOs (Figure S3C). We add additional detail on tonic vs. phasic REM substages in Supplementary Figure S3, showing that the rate of HFOs and HFO chains is significantly elevated in putative phasic REM. While we would have liked to analyze REM HFOs during tonic and phasic states separately to be able to make more concrete conclusions, the scarcity of phasic REM sleep made it difficult to make accurate comparisons between the two, especially for spiking modulation. We have therefore removed the qualifying term “phasic REM” from the abstract.

Also, we would like to clarify that while we investigated chains of events (ripples) in NREM sleep as a comparison to REM HFOs, we do not refer to them as HFOs in NREM anywhere in the manuscript. In NREM, while there are PFC ripples that are clustered into chains based on our definition (separation of <200 ms), we do not observe a prominent peak in the IEI distribution that would suggest entrainment by other oscillations (e.g. theta or spindles). This analysis was only to show that associated results are specific to REM HFO chains, and not seen during comparable chains of NREM cortical ripple events.

Chain vs isolated HFOs: Regarding the Reviewers comment about why we chose to compare isolated and chain HFOs, the motivation is clearly demonstrated by differences in these events in spectral properties (Figure 3H-I) and spiking modulation (Figure 4F-G). Our initial motivation to investigate these chains of events in REM sleep came from our observation that PFC population activity aligned to REM HFOs was theta-modulated (multipeaked, suggesting multiple high-frequency events over a short duration). This led us to hypothesize that there may be chaining of events in REM sleep, which in line with previous studies demonstrating the clustering of events, such as spindles in NREM sleep (Darevsky, Kim et al. 2024). In that study (Darevsky, Kim et al. 2024), the authors showed that reactivation of motor patterns was more persistent during trains of spindle events as compared to isolated spindles. Furthermore, trains/chains of multiple hippocampal SWRs have been shown to underlie the replay of extended experience (Davidson, Kloosterman et al. 2009). Thus, the separation of high-frequency events into isolated and chained events has precedence and may have functional significance. Furthermore, since we see that isolated and chain events have a bias for occurring during putative bouts of tonic and phasic REM sleep, respectively (Figure S3C), characterization of both event types is an important step for understanding potential differences in interregional interactions during REM sleep. Furthermore, a recent appreciation for the role of sleep stage sub-states (Chang, Tang et al. 2025) further emphasizes the importance of investigating these events separately. We expect future studies to further dissect the roles of tonic and phasic REM states in memory and cognition, and our finding provides an account of the existence of different events for future reference.

HFO chains during periods of greater synchrony: While it may seem natural that chaining would occur during periods of high synchrony (theta synchrony here), we show that our result is HFO-specific, especially for spiking activity modulation (Figures 4F-H and Supplementary Figures S5K-N), which makes it novel and important. Previous studies have focused on theta-gamma cross-frequency phase amplitude coupling, which has been proposed as a mechanism where slower theta oscillations temporally organize faster local population activity, thereby synchronizing neural ensembles within and across brain regions (Belluscio, Mizuseki et al. 2012). Here, we present a comparison of HFOs and gamma events and show that while gamma events can also occur in chains (added to Supplementary Figure S4H), possibly due to increased synchrony as the Reviewer stated above, we do not observe theta-modulated population activity aligned to these events (Supplementary Figure S4J), which is a defining property of HFOs that we propose underlies our results. This indicates that our reported results are unique to periods of synchrony associated with HFOs.

Control analyses for theta periods: Regarding the comment about lack of control for sleep state, we refer the Reviewer to our response to Reviewer 1’s comments. We have performed additional control analyses comparing PFC activity during REM theta periods adjacent to detected HFOs and demonstrate that phasic PFC population activity is largely absent (Figure S5).

We are aware that it is difficult to dissociate the generation of ripples and HFOs from the underlying brain state, since they are so tightly linked. However, we do expect that our clarifying points in addition to the REM theta state controls provide strong evidence that the results that we present are intrinsic to HFOs and not general reflections of activity during baseline theta activity in REM. Here, we further reiterate a few of the main results that demonstrate this:

(1) Phasic spiking modulation of PFC population activity associated with HFOs is not present during baseline theta periods (Supplementary Figure S5K).

(2) Phasic spiking modulation during HFOs is not linked to extracted gamma events (Supplementary Figure S4J).

(3) Phasic spiking modulation, as well as activity suppression, is strongest during chains of HFOs (Figure 4F, Supplementary Figure S4J).

(4) PFC-CA1 theta coherence surrounding HFOs increases relative to baseline (Figure 3E, z-scored relative to baseline coherence).

(5) Assembly peaks are sequentially organized surrounding HFO chains but not during isolated or shuffled (baseline) HFO times (Figure 6C, Supplementary Figure S7E, and Author response image 2).

(6) A higher proportion of CA1-CA1 pairs are high cofiring during HFOs as compared to baseline periods (Figure 7B), indicating specific CA1 engagement during HFOs (in addition to coherence).

Lastly, we show that HFOs detected during periods of active behavior (high theta) on the W-Track are not associated with many of the defining features of HFOs in REM sleep, thus demonstrating the specificity of REM HFOs despite similar background theta activity (Figure S11, in response to Reviewer 3 comment #1).

(2) It is crucial that the study provides REM-specific sleep examples for each of the data sessions, marking tonic and phasic REM and indicating when isolated and chain HFOs are observed. It remains unclear how interspersed these events are. Do some REM episodes just have isolated HFOs and others chains?

We thank the Reviewer for raising this point and apologize for the lack of clarity. For additional transparency, we now provide example sleep state plots for each animal (Supplementary Figure S1H-I).

We also provide hypnograms that show bouts of putative phasic REM on top of REM periods (Supplementary Figure S3). Additionally, as a compact way of demonstrating the validity of our separation of putative tonic and phasic bouts, we provide a plot showing the average velocity and spectrogram surrounding putative phasic REM bouts across all putative phasic REM transitions (Supplementary Figure S3A). Regarding the incidence of HFOs in tonic and phasic REM, we refer the Reviewer to Supplementary Figure S3D, where we show that HFO rate is significantly higher during putative bouts of phasic REM, during which they tend to be organized in chains as compared to putative tonic bouts (Supplementary Figure S3E). In line with this, we additionally report that although both isolated and chain HFOs occur in putative tonic and phasic bouts of REM sleep, the proportion of chain HFOs during phasic REM is significantly higher than that of isolated HFOs (Figure S3), indicating a bias for chains to occur in phasic REM sleep, potentially due to stronger theta input. However, since putative phasic REM accounts for <10% of REM sleep in our dataset, consistent with previous reports using similar methods (Mizuseki, Diba et al. 2011), we were unable to restrict spiking analysis to HFOs in phasic REM. We found that chain HFOs during both putative tonic and phasic REM sleep elicited theta modulated population activity in PFC, thus we pooled HFOs across REM states for analysis.

While a larger proportion of chain events occur in putative bouts of phasic REM sleep as compared to isolated events (Figure S3), chain and isolated events occur in both putative tonic and phasic substates (Proportion of events in putative tonic REM is (1 - proportion in phasic) shown in Figure S3C, right). Thus, the majority of analyses comparing isolated and chain HFOs, especially for spiking data, were pooled across states. Instead of showing example plots for all 22 sleep epochs (22 out of 36 epochs with >5 s of putative phasic REM), we expect that this analysis will be sufficient to illustrate the distributions of isolated and chain HFOs across putative REM sleep substates. We have also removed the qualifying term “phasic REM” from the abstract.

To clarify this, we have added a statement to the new “Limitations” section of the manuscript on Page 16, Lines 10-20:

“Third, we did not record eye movements or ponto-geniculo-occipital (PGO) waves, both of which would have allowed for more accurate segregation of tonic and phasic REM sleep states. (Simor, van der Wijk et al. 2020) Although we observed a bias for isolated and chain HFOs to occur in putative tonic and phasic REM substates, respectively, the scarcity of putative phasic REM bouts made the direct comparison based on substage difficult. Finally, although our model predicts that distinct cell-type activity profiles shape REM sleep HFO dynamics, we did not record a suDicient number of interneurons to test these predictions directly. Future studies using appropriate behavioral tasks, longitudinal sleep recordings, and cell-type specific opto-tagging will be able to resolve these limitations and further clarify the roles of high-frequency oscillations in REM sleep.”

We have now added Supplementary Figure S3.

This is also important because some of the REM sleeps detected and shown in Figure S1H seem unusual. For example, in Animal 1, there are multiple bursts of REM that seem very irregular. In some other sessions, animals occasionally appear to enter REM sleep with very little preceding NREM, which goes against existing literature. It's not clear which ones of these meet the 30 s duration threshold. Are the chain events occurring in these periods?

In reference to the hypnograms shown in Supplementary Figure S1H (Now Supplementary Figure S1I), these were generated by concatenating all 9 sleep epochs regardless of whether they passed the inclusion criterion of > 30 s of total REM sleep. Furthermore, only a subset of the epochs shown were included for analysis based on a secondary, manual inspection that is performed to confirm inclusion. Sleep state plots (e.g. Supplementary Figure S1H) were visually inspected to further confirm the transition into REM sleep, ensuring absence of noisy T/D ratio or spurious detection due to noisy signals – epochs where microarousals or persistent subthreshold fluctuations in animal movement induced noisy TD ratio increases, and thus inaccurate REM designation, were excluded. We thus used a total of 36 sleep sessions from a possible 90 sleep sessions.

We apologize for not specifying what portions of data represented by the hypnograms were included. We have now provided updated hypnograms only illustrating the sleep epochs included for analysis (Figure S1I).

We have now added Supplementary Figure S1.

Regarding the Reviewer’s point “Are the chain events occurring in these periods?”: Yes, all of the chain events (and isolated events) come from these updated epochs that are now shown. No events in the excluded epochs were included, since we wanted to only analyze the data that came from curated REM epochs that we were confident in.

(3) The analysis supporting structured reactivation was not generally convincing. Figure 4E does not provide convincing evidence of this. Indeed, reactivation strength is lowest around the time of the event, and appears highest 0.5s before. The example panel seems rather anecdotal. It's also not clear why REM reactivations should be compared to NREM ones here. I could not follow what was done in Figures 4F-H. Why should the first half and second half of an HFO event be correlated?

We appreciate the Reviewer for raising these concerns. We agree that this section is somewhat dense and at time hard to follow, so we will clarify with further explanations and analyses (see also response to Comment #8 with new reactivation figures).

In Figure 6B (originally Figure 4E), our intention was to show that if you simply align assembly activation to REM HFOs, a relatively flat response is observed when averaged across all assemblies, in stark contrast to assembly reactivation during NREM cortical ripples. This could suggest a couple of things: (1) There is no real assembly activation in response to REM HFOs or (2) Assembly activation is organized differentially (compared to NREM) surrounding HFOs. What we hypothesized, and then quantified based on observations, is that assembly activity is sequentially organized around HFOs. If this were true, it would suggest a consistent temporal relationship between REM HFOs and assemblies (for example, assembly 1 tends to be active 115 ms after the onset of chains, assembly 2 is active 230 ms after, etc.). Of course, the sequential pattern that we show in Figure 6A may arise trivially, especially since these types of sequential visualization plots can simply arise from noise. Thus, a cross-validation method must be utilized to ensure the sequences are indeed reflective of an underlying computation.

In the methods section “Assembly sequence detection surrounding HFOs” we explain the splitripple/HFO procedure that we used to compare assembly sequences across two halves of the data, similar to methods used in hippocampal place cell sequence cross-validation (Plitt and Giocomo 2021, Sosa, Plitt et al. 2025). For every split and assembly alignment, a sequence similar to Figure 6A, right is generated based on the first half of aligned data. Then the second half of aligned data is sorted based on the peak indices of the first half of data and the correlation between the peak reactivation indices across all assemblies for the two datasets is calculated. A high correlation indicates high sequence similarity across the two halves of data (not two halves of an HFO as the Reviewer mentioned), suggesting temporal consistency of assembly reactivation. By utilizing this method, we show that assembly reactivation sequences across randomly chosen halves of data are most similar for chained HFOs (Figure 6C and Supplementary Figures S7C-F). We have now provided an additional control analysis that investigates this sequential assembly activity for time-shifted chain events (Author response image 2). As in Figure 6, assembly activity was aligned to the first event in each time-shifted chain. In addition to the analyses in Supplementary Figure S7, this control further indicates that sequential assembly activity is preferentially restricted to HFO chains.

**Author response image 2.** Assembly sequences surrounding time-shifted HFO chains. (A) Distributions of r values calculated from the Pearson correlation between peak reactivation bins across all assemblies for two randomly chosen halves of the shifted HFO-aligned data. There was no difference between r values for time-shifted chains and shuffled data, indicating no structured assembly activity during periods outside of real HFOs chains.

To further quantify this, we calculated two additional metrics: (1) the slope difference between fitted lines for the two halves of data for each split and (2) the absolute peak difference between assemblies in the two halves of data. First, for the slope difference metric, the slope of the best-fit line between assembly ID and peak reactivation index was taken for the two halves of data and compared. A smaller slope difference compared to shuffled data in Figure 6D indicates that assembly reactivation is structured in a more similar manner across the two halves of the real data. Secondly, Figure 6E is a quantification of the peak reactivation displacement between the two halves of data. If there is a high probability of small peak differences, as in the real data, this indicates that the timing of peak reactivation of assemblies relative to HFO chain onset is similar across the two halves of data.

We apologize for the omission of the description of the slope and peak difference metrics that we used in Figures 6D,E. We have added information in the Methods section under “Assembly sequence detection surrounding HFOs” on Page 43, Lines 23-33:

“Furthermore, the slope and peak differences were calculated as additional metrics of sequence and temporal reactivation consistency between the two halves of data, respectively. For the slope difference metric, the slope of the best fit line between assembly ID and peak reactivation index was taken for the two halves of data and compared. A smaller slope difference compared to shuffled data indicates that assembly reactivation is structured in a more similar manner across the two halves of the real data. For peak reactivation difference, the temporal displacement of the peak reactivation index between the two halves of data was calculated and compared to shuffle. A high probability of small peak differences indicates that the timing of peak reactivation of assemblies relative to HFO chain onset is similar across the two halves of data. Shuffling of assembly strength was carried out as above.”

(4) The authors argue that the occurrence of HFOs, rather than theta power, is the reason for lower MUA activity, but the analysis for this (Figure S4I) is quite confusing. The left panel actually seems to indicate that MUA is indeed lower when theta power is high.

We apologize for the confusion regarding this figure and appreciate the Reviewer’s point that periods of high theta power can also appear to be associated with reduced MUA. We agree that the original presentation may not have clearly separated the contributions of theta and HFOs. What we convey with Supplementary Figure S5K (originally Supplementary Figure S4I) and new Supplementary Figures S5L-M is that the observed theta-modulated PFC spiking response (Figure 2A) cannot be solely explained by baseline theta periods (outside of HFOs) in REM sleep. Since theta oscillations are ubiquitous during REM sleep, an important control is to demonstrate that the theta-modulated population activity is not simply a consequence of ongoing theta activity. Thus, we aligned PFC activity to theta oscillations of varied power and show that the fluctuating, theta-modulated population activity is absent, indicating that HFOs associated with the theta oscillation are driving this phasic response.

We are not solely arguing that the presence of HFOs is the driver of decreased PFC multiunit activity. In both the data and model, we show that the magnitude of theta power detected in PFC (possibly input to PFC) is inversely related to multiunit activity (Figure 9E). Our interpretation is not that theta is unrelated to MUA, but rather that HFO occurrence provides additional explanatory power beyond theta alone. Accordingly, we show directly in Supplementary Figures S5L-M, in response to Reviewer 1’s comment #1, that theta periods in phasic REM not associated with HFOs do not elicit the MUA activity suppression similar to HFOs.

The phase-alignment performed is hard to follow and is not being applied to HFO periods. If the study is trying to argue that high-theta periods without HFOs in the same recording sessions show lower MUA, then perhaps some sort of shuffle or jitter would be more suitable. For example, in Figure 1B, it seems there are some high-theta periods that don't have HFOs and appear to have higher MUA.

We expect that the new analyses where we provide additional baseline theta controls for periods adjacent to HFOs in Supplementary Figures S5L-M now clarifies this point. Briefly, alignment to theta phases at different temporal distances from detected HFOs does not exhibit the same fluctuating PFC activity as HFO alignment.

Regarding the phase alignment procedure that we used for the control analysis, since REM sleep is characterized almost entirely by ongoing theta activity, the control condition was not a separate brain state but rather theta periods outside of HFOs. We therefore needed a systematic way to select comparable theta cycles and phase bins in order to make a valid comparison with HFO-aligned PFC population activity. Since we demonstrated that there is significant phase amplitude coupling between theta and HFOs, thus a theta phase preference of HFOs, we used that specific phase bin across multiple theta cycles to align PFC activity. This phase bin selection was performed separately for each epoch to account for inter epoch and animal variability in phase preference. We reasoned that this procedure would allow for a valid comparison as compared to random alignment, since activity was aligned to similar phases in the baseline theta vs HFO conditions.

(5) As far as I could tell, the study does not distinguish between putative excitatory and inhibitory neurons in the PFC, but only in the CA1, even though these play very different roles in the model. What is the rationale for not separating these? How are reactivations to be interpreted among interneurons?

We apologize for the lack of emphasis on this point, which was originally highlighted in Supplementary Figure S2G (now in Supplementary Figure S2F), and for omitting the explanation as to why we did not separately analyze putative excitatory and inhibitory neurons. When we plot the average waveform peak-to-trough and mean firing rates of the PFC neurons, we observe a large cluster with moderate mean firing rates and peak-to-troughs consistent with recording primarily from pyramidal neurons (Supplementary Figure S2F, left). We therefore decided to pool and not separate the populations into putative pyramidal cells and interneurons for the spiking analyses presented. To further validate our decision to pool the cells, we separated the population into putative pyramidal cells and interneurons based on peak-to-trough. Putative interneurons were identified as cells with a peak-to-trough <0.3 ms (we obtained similar results when using a hyperplane to separate units based on both peak-to-trough and firing rate, with a smaller subset identified as putative interneurons). When these putative interneurons were excluded from the HFO-aligned multiunit PFC plot, we observed very similar activity to Figure 2A (Supplementary Figure S2F, right). We thus decided to pool the cells into a single population for the purpose of this manuscript, as we did not have enough interneurons to investigate them separately. We are, however, aware that different cell types may contribute to the phenomenon that we report here and attempt to more thoroughly differentiate the contribution of pyramidal cells and interneurons with our modeling result in Figures 9 and 10.

We have now added the following to the figure legend on Page 51, Lines 21-24:

“REM HFO aligned PFC multiunit response when spikes from putative interneurons are excluded (compare to Figure 2A). Due to this similarity of the phasic PFC response when putative interneurons are omitted, we decided to pool PFC neurons for all further analyses.”

Regarding the interpretation of reactivation in the context of interneurons, we expect reactivation reflects coordinated ensemble activity with excitatory neurons encoding task-relevant information, and inhibitory interneurons shaping timing and neural synchronization. We however did not record enough distinct interneurons to test the predictions of the model, which is now noted in the Limitations on Page 16.

Relatedly, we find that PFC assemblies detected from pooled data have task relevant representations (Figures 5F-G).

(6) Are the high-cofiring CA1 neurons generally higher-firing than the other cells? Could this perhaps explain why they behave differently?

We apologize that this information was not more evident in the manuscript, as it is an important control. In Supplementary Figure S9A, we show that there was no difference in baseline firing rate between low and high cofiring CA1 neurons.

We have now explicitly referenced this figure in the main text on Page 9, Lines 42-45:

“Analysis of low and high cofiring CA1 neurons during REM HFOs showed that high cofiring neurons exhibited elevated activity during chained events as compared to low cofiring neurons (Figure 7D), independent of baseline firing rates (Figure S9A).”

(7) It appears that the decreased firing around HFO's could be a consequence of the stronger firing modulation around these periods, related to time averaging, rather than suppression per se. How does the firing rate compare to other periods with similar modulation that might not have HFOs?

We thank the Reviewer for raising this important point. We expect that the new analyses, where we provide additional baseline non-HFO-associated theta periods, and theta periods adjacent to HFOs at different temporal distance as controls in also Supplementary Figure S5L-N now clarifies this point. These figures show that the decreased firing rate is specific to HFO chains (Supplementary Figure S5N). Indeed, if the suppression that we observe is related to time averaging, or another analytical artifact, our claims of suppression during HFOs would not be valid. We present a number of results and provide further explanations to support the accuracy of our characterization of PFC population suppression.

First, event-aligned multiunit activity was quantified as baseline-normalized population firing relative to detected events. For both NREM and REM, activity was normalized by the mean population firing rate during a baseline period within the same sleep state in which events were detected. Values are therefore expressed as deviations from baseline. This normalization allows comparison of relative changes in firing around events within each state. Importantly, values below baseline reflect reductions relative to the state-matched baseline period.

Second, we show that smoothing activity with a larger gaussian kernel preserves the dip in population activity, consistent with suppression of activity surrounding HFOs (Figure 9C, note that this is a data figure presented in the context of the model). However, as raised by the Reviewer, this normalized measure does not on its own distinguish sustained suppression from transient deviations introduced by event-locked temporal structure in firing.

Third, to address this, we employed an alternative method to demonstrate that HFO chains tend to occur during periods of PFC suppression (Figure 4G). Briefly, we detected events in PFC where activity fell below a threshold and calculated the probability of HFOs surrounding these “suppression” events. We refer the Reviewer to the Methods section under “Detection of population suppression” where we explain this procedure in more detail. We found that chain ripples, during which the strongest suppression is observed (Figure 4F), are associated with decreases in PFC activity (Figure 4G and Supplementary Figure S6).

Fourth, we refer the reviewer to Figure S5N, where we compare the firing rates of PFC neurons during HFO chains and theta periods outside of HFOs during putative phasic REM bouts. The observed reduction in firing during true HFO chains compared to non-HFO periods therefore reflects HFO event-specific activity suppression rather than a common occurrence during baseline REM periods.

Lastly, we performed a control analysis complimentary to Supplementary Figure S5K where we aligned PFC activity to the preferred theta phase of HFOs and investigated how distance from detected HFOs modulates PFC activity (Figure S5). We found that there was no consistent theta-modulated activity aligned to HFO-adjacent theta phases.

Regarding the final comment, if the Reviewer meant “modulation” as in the theta modulation or suppression observed when PFC population activity is aligned to HFOs (Figures 2A and 4F), we are not aware of any other REM periods where this strong theta modulation or suppression of PFC population activity is present. To our knowledge, we are the first to demonstrate such a modulation of PFC population activity in REM sleep. The closest comparison that we can make is PFC activity aligned to gamma events that are coordinated with HFOs (suppression of PFC), but this is explained only with association with HFOs, as shown in Supplementary Figure S4J.

(8) The assembly reactivation measure does not control for pre-existing assemblies. The term "activation strength" would therefore be more appropriate.

We thank the reviewer for this important methodological point. The concern that ICA-based reactivation strength does not, by itself, distinguish behavior-induced reactivation from pre-existing assembly activity/structure is well-taken, and we have implemented several complementary analyses that directly address these concerns.

First, the interleaved structure of our recordings (8 run epochs interleaved with 9 sleep epochs) allows us to investigate the within-session pre/post assembly strength differences (i.e. each W-Track run session has a preceding (pre) and following (post) sleep session). An increase in assembly strength from pre to post is a hallmark of behaviorally relevant assembly reactivation (Kudrimoti, Barnes et al. 1999, Peyrache, Khamassi et al. 2009). For each run epoch, the same run-derived templates were projected onto the preceding and following sleep epochs, and the pre and post strengths were compared. The distribution of post-minus-pre reactivation differences across all epoch pairs is significantly skewed toward positive values (Figure 6F), indicating that templates from the run epochs are more strongly expressed in the post-sleep epochs. This asymmetry cannot be explained by pre-existing assembly structure, which would predict similar assembly strengths.

Second, reactivation strength in post-experience sleep increases across the experiment, with templates from later running epochs producing the strongest reactivation in the following post-sleep (Figure 6G). This increase in reactivation strength over time cannot be explained by preexisting assembly structure, which predicts similar assembly expression strength independent of experience.

Third, the detected assemblies carry behaviorally meaningful structure. Assembly activation maps computed during running exhibit spatially organized "assembly fields" similar to single-cell place fields (Figure 6H), demonstrating that the detected assemblies represent specific spatial locations or task variables rather than behavior-independent states. Pre-existing co-firing structure unrelated to ongoing experience would not be expected to produce spatially tuned, task-locked assembly activation. Furthermore, this spatial tuning of assemblies was verified by comparison with surrogates, where assembly maps were generated using circularly shuffled activation times (1000 shuffles). Assemblies with p < 0.05 (z > 1.65) were considered to have significant spatial structure (Figure 6I).

These results establish that what we measure is the selective re-expression of behaviorally relevant assemblies in subsequent sleep epochs, consistent with the use of "reactivation" in the established literature (Peyrache, Khamassi et al. 2009, Lopes-dos-Santos, Ribeiro et al. 2013). We have therefore retained the term "reactivation strength" and have added text to the manuscript noting these new results that justify the use of “reactivation” strength.

We have now added Figure 6.

We have also added the procedure for the calculation of spatial information to the Methods section under “Spatial information of assembly fields” on Page 44, Lines 8-23.

(9) Can the study rule out that the rank-ordering in Fig 4C is related to firing rates? Higher-firing rates tend to fire earlier, and lower-firing cells later.

We thank the Reviewer for raising this interesting point. Here, we assume that the Reviewer meant the baseline firing rates of the neurons, not the intra-HFO firing rates of the neurons. Indeed, when we look at baseline REM firing rates of these PFC neurons, we do find that neurons with higher firing rates tend to fire earlier than low-firing-rate neurons (Author response image 3). This is also true when PFC rank and firing rate are assessed for isolated REM HFOs and NREM PFC ripples (Author response image 3). Similarly, we also observe this relationship in CA1 during SWRs, during which rank order correlation is typically assessed as a method for replay detection. In line with this, a previous study has shown that CA1 neurons with high excitability at animals’ current location tend to initiate replay events (Karlsson and Frank 2009). Furthermore, high-firing-rate, rigid CA1 neurons are more active during SWRs than low-rate, plastic neurons (Grosmark and Buzsaki 2016), and there are distinct populations of neurons in both hippocampus and PFC that are preferentially active during immobility in sleep epochs (Jarosiewicz, McNaughton et al. 2002, Kay, Sosa et al. 2016, Tang, Shin et al. 2017), potentially biasing replay activity during high-frequency events. Similar dynamics may underlie activity during PFC ripples and HFOs in NREM and REM sleep, respectively. The critical point here is in the leave-one-out cross-validation that we implemented to determine sequence similarity—each left out event’s cell rank was correlated with the averaged rank of the template that was generated from all other events. This analysis provides a basis for our claim that there is preserved sequential PFC activity across HFO chains. We did not observe neuron firing consistency during isolated HFOs or during pseudo-HFO chains (coherently shifted chain HFO times), which indicates that REM HFO chains are unique temporal windows during which PFC activity proceeds in a more structured manner.

**Author response image 3.** Firing rate difference of low and high rank neurons. (A) Comparison of baseline firing rates of PFC and CA1 neurons split by average rank across all PFC REM HFOs, PFC NREM ripples, or CA1 SWRs. Baseline rates were calculated separately for NREM and REM sleep.

Minor:

(1) It gets confusing that the authors sometimes (but not always) refer to HFOs during NREM as "ripples" but not if they occur during REM. The terminology is inconsistent. When they refer to HFO chains, it seems they now pool between REM and NREM periods, as well as across phasic and tonic REM periods, which is confusing.

We apologize for the confusion regarding the terminology. In the revised manuscript, we now use NREM ripples exclusively for NREM events and REM HFOs exclusively for REM events. We have removed mixed labels such as “ripple/HFO” except where a collective term is explicitly defined. We also clarified that HFO chains refer to REM events only and revised the relevant text/figure legends to avoid any implication that chain analyses pool NREM and REM events.

(2) P7 L8: It might be helpful to emphasize "broader temporal distribution".

We thank the Reviewer for the suggestion. We have updated the text on Page 8, Line 18:

“Since we observed a broader temporal distribution of activity…”

(3) P8 L9: What do they mean by spatial? Do they mean the place-fields of these same neurons during a previous task period?

We apologize for the confusion. The Reviewer is correct. Here, we calculated the spatial rate map correlation between CA1 neurons as a measure of place field similarity during the W-Track session prior to the sleep epoch being assessed.

For clarification, we have added “rate map” to the text on Page 9, Line 33:

“…spatial rate map correlation…”

We have also updated the y-axis label for Figure 7B for clarity.

(4) P8 L27: What do they mean by "coordinated SWRs"? As opposed to what?

Here, we are referring to our previous study where we investigated ripples in NREM sleep and showed that ripples and SWRs in PFC and CA1, respectively could either be independent from or coordinated with events in the other region (Shin and Jadhav 2024). A main result in the study showed that CA1 neurons are strongly suppressed during independent PFC ripples and that there was a relationship between activity suppression and reactivation during coordinated SWRs (CA1 SWR-PFC ripple coordination in NREM). We specifically mentioned “coordinated” since these are SWRs that are also coupled with SOs and spindles as compared to SWRs that are independent from PFC ripples (Shin and Jadhav 2024). Overall, we wanted to frame this result in the context of oscillatory coupling and mechanisms of memory consolidation.

(5) P37 L28 says "we observed a bimodal distribution" but L31 says "unimodal". Which is it?

We apologize for the confusion. We observed a bimodal distribution for CA1-PFC cofiring in REM sleep, but a unimodal distribution in NREM sleep. Because of these two observations, we decided to split the CA1 population into high and low cofiring neurons based on two different thresholds:

(1) Splitting the population by cofiring values greater than (high cofiring) or less than (low cofiring) 0.

(2) Splitting the population by cofiring values greater than (high cofiring) or less than (low cofiring) the mean of the distribution of averaged cofiring values.

Using two separate thresholds to split high and low cofiring CA1 neurons demonstrates the robustness of the firing rate change result in Figures 7F and Supplementary Figures S9B-D.

We added a statement that clarifies that the bimodal distribution was seen in REM sleep only on Page 44, Lines 37-43:

“Furthermore, since we observed a bimodal distribution of CA1-PFC cofiring values in REM sleep, we split the population based on whether the average (across all cell-cell combinations) cofiring value or correlation coefficient of a cell was above or below 0. Also, since the NREM ripple cofiring distribution was unimodal, we additionally split the populations using the mean of the average cofiring or correlation coefficient distributions.”

**Reviewer #3 (Public review):**

Summary:

Shin et al. examine hippocampal-prefrontal interactions during sleep using simultaneous CA1 and prefrontal cortex recordings in rats performing a spatial memory task. They identify high-frequency oscillation (HFO) events in PFC during REM sleep that occur in theta-modulated chains and are associated with increased CA1-PFC coherence and sequential, sparse reactivation of cortical ensembles. This pattern contrasts with the synchronous reactivation observed during NREM cortical ripples. Together with a simple cholinergic network model, the authors propose that REM HFO chains represent a distinct mechanism for hippocampal-cortical coordination that complements NREM ripple-mediated processing during sleep.

Strengths:

A major strength of the work is the extensive electrophysiological dataset, which includes simultaneous recordings of large neuronal populations in both hippocampus and prefrontal cortex across behaviour and subsequent sleep. The analyses linking high-frequency events to population dynamics, interregional coherence, and ensemble reactivation are technically sophisticated and provide an incredibly detailed description of REM-associated cortical activity patterns. In particular, the demonstration that REM HFOs occur in chains aligned to theta phase and organise sequential activation of cortical assemblies represents a potentially important advance in understanding the neural structure of REM sleep activity. The integration of experimental data with a computational model further provides a useful framework for interpreting the observed differences between REM and NREM network states in terms of neuromodulatory influences.

Weaknesses:

While overall this study provides a highly valuable body of work, there are two primary limitations, which, if overcome, would provide substantially more significance to the overall characterisation of REM HFOs. Specifically:

(1) Distinction from wake HFOs

The results largely support the authors' claim that REM HFO chains represent a distinct pattern of neural coordination compared to NREM cortical ripples. The analyses consistently show differences between REM and NREM events in terms of neuronal modulation, ensemble structure, and interregional coupling. However, similar high-frequency events during wake are not examined. Since REM sleep shares several network features with wakefulness, including strong theta oscillations, evaluating whether comparable PFC HFOs occur during wake would provide clarity on whether these events are specific to REM sleep (and its associated functions) or represent a more general theta-associated phenomenon.

To investigate PFC high-frequency oscillations during running behavior on the W-Track, events were extracted in the same manner as NREM and REM events (Methods). Events during wake were subset by periods where the animals’ velocity was >4 cm/s to provide a comparison of events during periods of high theta. While we were able to detect HFOs during wake that exhibited a similar spectral profile in the high frequency band, we did not observe (1) strong association with gamma or theta oscillations, (2) prominent HFO chaining, (3) HFO aligned theta modulated PFC activity, (4) comparable levels of theta phase amplitude coupling, (5) association with population suppression, or (6) a relationship between peri-event theta power and multiunit activity (Supplementary Figure S11). Many of the defining features of PFC REM HFOs are absent during wake, indicating REM specificity of the results we present.

(2) Link to memory consolidation

The manuscript proposes throughout that REM HFO chains may contribute to memory consolidation by coordinating hippocampal-cortical reactivation, but the evidence for this functional role remains indirect. The authors do highlight this as a limitation of the study - the inability to link their findings to learning - but it is not clear why. Further details of the behaviour results should be included. If no learning occurred across the eight behavioural sessions, this should be reported. If learning did occur, but could not be linked to HFO events, this should also be reported.

To address these concerns, we have now added an explicit “Limitations” section in the main text of the manuscript that includes a statement about learning. We have also added Supplementary Figure S1 in the manuscript, which illustrates the performance of all 10 animals on the W-Track task. Finally, we have also included Figures 6F G in the manuscript, showing that PFC assembly reactivation strength during sleep epochs increases during learning.

**Reviewer #3 (Recommendations for the authors):**

Most of my specific comments were related to further clarification that will help the reader's understanding.

(1) I'd recommend simplifying terminology. Open to debate, but would it not be simpler and clearer to just say NREM HFO vs REM HFO? Obviously, there is a need to mention how NREM HFOs have previously been referred to as cortical ripples, but I'm not sure it is such a helpful terminology to continue for the field, given, as you state, how different cortical ripples are from hippocampal SWRs. If not, I'd at least provide a clearer explanation early in the manuscript, distinguishing NREM ripples from REM HFOs but collectively still calling them 'cortical events'.

We appreciate the Reviewer’s suggestion regarding terminology and agree that it would be simpler and clearer to use a single term, ripple or HFO, to describe these events. Initially, we had used a unified term (ripples across both states) but ultimately decided to switch to state-specific terminology due to previous comments we received on the manuscript and to emphasize the distinctions between NREM and REM events. Ultimately, we decided on calling them ripples in NREM and HFOs in REM since there is precedence for both terms in each respective sleep state (Khodagholy, Gelinas et al. 2017, Vaz, Inati et al. 2019, Bueno-Junior, Ruckstuhl et al. 2023, Shin and Jadhav 2024), but we do agree that this distinction can be confusing if not clearly stated. Thus, we have added an additional statement in the manuscript on Page 4, Line 44 to Page 5, Lines 1-3 for clarity:

“Similar criteria were used to detect cortical high-frequency events in NREM and REM states; however, to avoid ambiguity and to conform to previous nomenclature, we refer to cortical NREM events as ripples, cortical REM events as HFOs, and hippocampal sharp-wave ripples in NREM as SWRs throughout”

(2) I assume experiments occurred during the light phase, but it would be good if this could be stated explicitly.

We have now added text specifying that these experiments took place in the light phase on Page 34, Lines 9-12 of the Methods section under “Behavior”:

“During the recording day, animals were introduced to the novel W-maze (~80 × 80 cm with ~7 cm wide tracks) for the first time and learned the task rules over eight behavioral sessions during the animals’ light phase between the hours of 9 AM and 6PM.”

(3) Page 2 Line 14: Rephrase to make clearer, e.g. 'that have a shift in...'.

We have rephrased the sentence for clarification on Page 2, Lines 13-15:

“REM HFO chains also preferentially engage CA1 neuronal populations that demonstrate a shift in their preferred theta-phase from behavior to REM sleep.”

(4) Page 4 Line 36 - 'and find coherent shifts in TD', this is self-fulfilling. I would rephrase to something like 'resulting in...'.

We have rephrased the sentence on Page 4, Lines 36-38:

“We separated NREM and REM sleep stages based on theta-to-delta (TD) ratio in CA1, which revealed coherent shifts in TD ratio across CA1 and PFC at the onset and offset of REM sleep…”

(5) Figure 1H left - clarify how many animals or multiunits this is based on.

We have now updated Figure 1 legend to specify the number of animals and epochs included on Page 19, Line 4:

“REM HFO aligned multiunit activity (MUA) in PFC (n = 10 animals, 36 epochs)…”

(6) Figure 1I (right), it would be useful to see the x-axis frequency start from 0, since you are cutting the peak in power.

We thank the Reviewer for this suggestion. We had initially set the frequency limits to 4 and 12 to specifically illustrate the absence of theta-modulated activity during NREM ripples. However, as suggested by the Reviewer, it is informative to expand the frequency range to ascertain the location of the peak frequency for NREM. Indeed, the peak frequency of NREM ripple-aligned PFC activity tends to be lower than 4 Hz, which is consistent with a single peak of activity that lasts <1 s.

We have now updated Figure 2C with these new panels.

(7) Figure 5 E/I - use of ** is confusing, it looks like a significance comparing e.g. quartile 2 to 1, but I think this is the correlation significance. I'd move ** to the top right corner and ideally include rho values.

We thank the Reviewer for this suggestion. We have now added the r values for the correlation to Figures 7E and 8B to resolve any ambiguity. In addition, we updated Figure 7F, right and Figure 5B to maintain consistency across main figures.

(8) Figure 7D - Why are stimulated and non-stimulated cells so different at baseline? This is not the case for the NREM results.

The y-axes in Figures 10C,D (Formerly Figure 7) show the fraction of cells with at least one spike per 10ms bin, either for all pyramidal cells or all interneurons. We report in the figure legend that the stimulated cells represent only 30% of the network for any given stimulus (Page 32, Line 18), so at baseline in both the NREM and REM simulations, there are roughly 3x as many non-stimulated cells with a spike per 10ms bin compared to stimulated cells, as these populations are active at roughly equal firing rates per neuron outside of stimulation periods.

(9) Methods - there is limited info on spike sorting procedure - can you provide a reference with further details (I couldn't find)? Is this method equally valid for identifying PFC units?

Matclust is a MATLAB-based spike sorting graphical user interface that allows for manual curation of neuron clusters through the visualization of spike waveform amplitude, peak-to-trough, and principal components. Polygons or boxes are drawn around spike data points, and single unit clusters are resolved through refinement in multiple dimensions. It was developed by Mattias Karlsson and was first used in a publication reporting replay of remote experiences in the hippocampus (Karlsson and Frank 2009). Although there is no formal reference, it can be found at https://bitbucket.org/mkarlsso/matclust/src/master/. Other labs have used the software for clustering neurons from cortical areas (Yu, Liu et al. 2018, Proskurin, Manakov et al. 2023), which demonstrates its robustness across multiple brain areas.

Additionally, examples of clustered neurons in PFC over the course of the experimental paradigm used here can be found in our previous publication (Shin, Tang et al. 2019). In addition to the aforementioned references, we show that PFC neurons can be accurately clustered and that neurons are stable over time, according to a number of cluster metrics.

(10) Why were there no further analyses of pyramidal cells and interneurons beyond Figure S2?

We thank the Reviewer for bringing up this important point, which is similar to Reviewer 2, comment #5 above. We repeat our response here. When we plot the average waveform peak-to-trough and mean firing rates of the PFC neurons, we observe a large cluster with moderate mean firing rates and peak-to-troughs consistent with primarily recording from pyramidal neurons (Supplementary Figure S2F, left). All results are similar if we exclude putative interneurons. To validate our decision to pool the cells, we separated the population into putative pyramidal cells and interneurons based on peak-to-trough. Putative interneurons were identified as cells with a peak-to-trough <0.3 ms (we obtained similar results when using a hyperplane to separate units based on both peak-to-trough and firing rate, with a smaller subset identified as putative interneurons). When these putative interneurons were excluded from the HFO aligned multiunit PFC plot, we observed very similar activity to Figure 2A (Supplementary Figure S2F, right). We thus decided to pool the cells into a single population for the purpose of this manuscript. We are, however, aware that different cell types may contribute to the phenomenon that we report here and attempt to more thoroughly differentiate the contribution of pyramidal cells and interneurons with our modeling result in Figures 9 and 10.

We have now added the following to the figure legend on Page 51, Lines 21-24:

“REM HFO aligned PFC multiunit response when spikes from putative interneurons are excluded (compare to Figure 2A). Due to this similarity of the phasic PFC response when putative interneurons are omitted, we decided to pool PFC neurons for all further analyses.”

(11) Looking at Figure S1B, the second to last main block of NREM sleep shown has a clear peak passing TD threshold, but oddly not classed as REM - I can only assume this is due to the duration limits on your classification?

Yes, this is due to the REM duration threshold that we implement in our sleep scoring algorithm. We used a minimum REM bout threshold criterion of 10 s for inclusion, as in previous reports (Rothschild, Eban et al. 2017, Zhang, Zhang et al. 2020). Additionally, we have included example sleep plots for all 10 animals in Supplementary Figure S1.

(12) Include details of how head speed was calculated - just based on the 30fps video?

Yes, the head speed of the animal was determined by tracking the animals’ position and calculating the speed based on cm/pixel values. We have now added more detail on this in the Methods section under “Surgical implant and electrophysiology” on Page 33, Line 44 to Page 34, Lines 1-2:

“Additionally, the animals’ speed was calculated based on predetermined cm/pixel values and the position displacement between frames captured at 30 fps.”

(13) Page 31 line 7 - With the reference you cite, they didn't really show tonic and phasic REM can be segregated based on theta frequency - they just defined it as such. You've done it for some, but I'd ensure all references to phasic REM are defined as putative - mostly missed within the discussion - I'd also add this as a brief limitation (without recording of eye movements or PGO waves).

We thank the Reviewer for these suggestions. We have now ensured that all mentions of “phasic REM” are qualified with “putative” and have added the requested limitation in the Limitations section on Page 16, Lines 10-15:

“Third, we did not record eye movements or ponto-geniculo-occipital (PGO) waves, both of which would have allowed for more accurate segregation of tonic and phasic REM sleep states.(Simor, van der Wijk et al. 2020) Although we observed a bias for isolated and chain HFOs to occur in putative tonic and phasic REM substates, respectively, the scarcity of putative phasic REM bouts made the direct comparison based on substage difficult.”

We have also modified the wording in the Methods section under “Theta inter-peak intervals during bouts of high and low theta power” on Page 37, Lines 14-15 to indicate that the referenced article simply used the theta frequency-based method to define tonic and phasic REM – not to explicitly separate the two states:

“Since previous studies have segregated putative tonic and phasic substages of REM sleep based on CA1 theta frequency…”

References:

Abdou, K., M. Nomoto, M. H. Aly, A. Z. Ibrahim, K. Choko, R. Okubo-Suzuki, S. I. Muramatsu and K. Inokuchi (2024). "Prefrontal coding of learned and inferred knowledge during REM and NREM sleep." Nat Commun 15(1): 4566.

Aleman-Zapata, A., R. G. M. Morris and L. Genzel (2022). "Sleep deprivation and hippocampal ripple disruption after one-session learning eliminate memory expression the next day." Proc Natl Acad Sci U S A 119(44): e2123424119.

Belluscio, M. A., K. Mizuseki, R. Schmidt, R. Kempter and G. Buzsaki (2012). "Cross-frequency phase coupling between theta and gamma oscillations in the hippocampus." J Neurosci 32(2): 423–435.

Bueno-Junior, L. S., M. S. Ruckstuhl, M. M. Lim and B. O. Watson (2023). "The temporal structure of REM sleep shows minute-scale fluctuations across brain and body in mice and humans." Proc Natl Acad Sci U S A 120(18): e2213438120.

Cairney, S. A., S. J. Durrant, R. Power and P. A. Lewis (2015). "Complementary roles of slow-wave sleep and rapid eye movement sleep in emotional memory consolidation." Cereb Cortex 25(6): 1565–1575. Chang, H., W. Tang, A. M. Wulf, T. Nyasulu, M. E. Wolf, A. Fernandez-Ruiz and A. Oliva (2025). "Sleep microstructure organizes memory replay." Nature 637(8048): 1161–1169.

Cheng, S. and L. M. Frank (2008). "New experiences enhance coordinated neural activity in the hippocampus." Neuron 57(2): 303–313.

Darevsky, D., J. Kim and K. Ganguly (2024). "Coupling of Slow Oscillations in the Prefrontal and Motor Cortex Predicts Onset of Spindle Trains and Persistent Memory Reactivations." J Neurosci 44(43). Davidson, T. J., F. Kloosterman and M. A. Wilson (2009). "Hippocampal replay of extended experience." Neuron 63(4): 497–507.

Ellenbogen, J. M., P. T. Hu, J. D. Payne, D. Titone and M. P. Walker (2007). "Human relational memory requires time and sleep." Proc Natl Acad Sci U S A 104(18): 7723–7728.

Foster, D. J. and M. A. Wilson (2006). "Reverse replay of behavioural sequences in hippocampal place cells during the awake state." Nature 440(7084): 680–683.

Ghosh, M., F. C. Yang, S. P. Rice, V. Hetrick, A. L. Gonzalez, D. Siu, E. K. W. Brennan, T. T. John, A. M. Ahrens and O. J. Ahmed (2022). "Running speed and REM sleep control two distinct modes of rapid interhemispheric communication." Cell Rep 40(1): 111028.

Grosmark, A. D. and G. Buzsaki (2016). "Diversity in neural firing dynamics supports both rigid and learned hippocampal sequences." Science 351(6280): 1440–1443.

Helfrich, R. F., J. D. Lendner, B. A. Mander, H. Guillen, M. PaD, L. Mnatsakanyan, S. Vadera, M. P. Walker, J. J. Lin and R. T. Knight (2019). "Bidirectional prefrontal-hippocampal dynamics organize information transfer during sleep in humans." Nat Commun 10(1): 3572.

Jarosiewicz, B., B. L. McNaughton and W. E. Skaggs (2002). "Hippocampal population activity during the small-amplitude irregular activity state in the rat." J Neurosci 22(4): 1373–1384.

Ji, D. and M. A. Wilson (2007). "Coordinated memory replay in the visual cortex and hippocampus during sleep." Nat Neurosci 10(1): 100–107.

Karlsson, M. P. and L. M. Frank (2009). "Awake replay of remote experiences in the hippocampus." Nat Neurosci 12(7): 913–918.

Kay, K., M. Sosa, J. E. Chung, M. P. Karlsson, M. C. Larkin and L. M. Frank (2016). "A hippocampal network for spatial coding during immobility and sleep." Nature 531(7593): 185–190.

Khodagholy, D., J. N. Gelinas and G. Buzsaki (2017). "Learning-enhanced coupling between ripple oscillations in association cortices and hippocampus." Science 358(6361): 369–372.

Kudrimoti, H. S., C. A. Barnes and B. L. McNaughton (1999). "Reactivation of hippocampal cell assemblies: effects of behavioral state, experience, and EEG dynamics." J Neurosci 19(10): 4090–4101. Lee, A. K. and M. A. Wilson (2002). "Memory of sequential experience in the hippocampus during slow wave sleep." Neuron 36(6): 1183–1194.

Leemburg, S., V. V. Vyazovskiy, U. Olcese, C. L. Bassetti, G. Tononi and C. Cirelli (2010). "Sleep homeostasis in the rat is preserved during chronic sleep restriction." Proc Natl Acad Sci U S A 107(36): 15939–15944.

Lopes-dos-Santos, V., S. Ribeiro and A. B. Tort (2013). "Detecting cell assemblies in large neuronal populations." J Neurosci Methods 220(2): 149–166.

Mizuseki, K., K. Diba, E. Pastalkova and G. Buzsaki (2011). "Hippocampal CA1 pyramidal cells form functionally distinct sublayers." Nat Neurosci 14(9): 1174–1181.

Nitsche, M. A., M. Jakoubkova, N. Thirugnanasambandam, L. Schmalfuss, S. Hullemann, K. Sonka, W. Paulus, C. Trenkwalder and S. Happe (2010). "Contribution of the premotor cortex to consolidation of motor sequence learning in humans during sleep." J Neurophysiol 104(5): 2603–2614.

Peyrache, A., M. Khamassi, K. Benchenane, S. I. Wiener and F. P. Battaglia (2009). "Replay of rule-learning related neural patterns in the prefrontal cortex during sleep." Nat Neurosci 12(7): 919–926.

Plitt, M. H. and L. M. Giocomo (2021). "Experience-dependent contextual codes in the hippocampus." Nat Neurosci 24(5): 705–714.

Proskurin, M., M. Manakov and A. Karpova (2023). "ACC neural ensemble dynamics are structured by strategy prevalence." Elife 12.

Rothschild, G., E. Eban and L. M. Frank (2017). "A cortical-hippocampal-cortical loop of information processing during memory consolidation." Nat Neurosci 20(2): 251–259.

Shin, J. D. and S. P. Jadhav (2024). "Prefrontal cortical ripples mediate top-down suppression of hippocampal reactivation during sleep memory consolidation." Curr Biol 34(13): 2801–2811 e2809.

Shin, J. D., W. Tang and S. P. Jadhav (2019). "Dynamics of Awake Hippocampal-Prefrontal Replay for Spatial Learning and Memory-Guided Decision Making." Neuron 104(6): 1110–1125 e1117.

Siapas, A. G. and M. A. Wilson (1998). "Coordinated interactions between hippocampal ripples and cortical spindles during slow-wave sleep." Neuron 21(5): 1123–1128.

Simor, P., G. van der Wijk, L. Nobili and P. Peigneux (2020). "The microstructure of REM sleep: Why phasic and tonic?" Sleep Med Rev 52: 101305.

Singer, A. C. and L. M. Frank (2009). "Rewarded outcomes enhance reactivation of experience in the hippocampus." Neuron 64(6): 910–921.

Sosa, M., H. R. Joo and L. M. Frank (2020). "Dorsal and Ventral Hippocampal Sharp-Wave Ripples Activate Distinct Nucleus Accumbens Networks." Neuron 105(4): 725–741 e728.

Sosa, M., M. H. Plitt and L. M. Giocomo (2025). "A flexible hippocampal population code for experience relative to reward." Nat Neurosci 28(7): 1497–1509.

Stark, E., L. Roux, R. Eichler and G. Buzsaki (2015). "Local generation of multineuronal spike sequences in the hippocampal CA1 region." Proc Natl Acad Sci U S A 112(33): 10521–10526.

Tang, W., J. D. Shin, L. M. Frank and S. P. Jadhav (2017). "Hippocampal-Prefrontal Reactivation during Learning Is Stronger in Awake Compared with Sleep States." J Neurosci 37(49): 11789–11805.

Tort, A. B., R. Scheffer-Teixeira, B. C. Souza, A. Draguhn and J. Brankack (2013). "Theta-associated high-frequency oscillations (110-160Hz) in the hippocampus and neocortex." Prog Neurobiol 100: 1–14.

Valero, M., T. J. Viney, R. Machold, S. Mederos, I. Zutshi, B. Schuman, Y. Senzai, B. Rudy and G. Buzsaki (2021). "Sleep down state-active ID2/Nkx2.1 interneurons in the neocortex." Nat Neurosci 24(3): 401–411.

van de Ven, G. M., S. Trouche, C. G. McNamara, K. Allen and D. Dupret (2016). "Hippocampal Offline Reactivation Consolidates Recently Formed Cell Assembly Patterns during Sharp Wave-Ripples." Neuron 92(5): 968–974.

van der Helm, E. and M. P. Walker (2011). "Sleep and Emotional Memory Processing." Sleep Med Clin 6(1): 31–43.

Vaz, A. P., S. K. Inati, N. Brunel and K. A. Zaghloul (2019). "Coupled ripple oscillations between the medial temporal lobe and neocortex retrieve human memory." Science 363(6430): 975–978.

Wilson, M. A. and B. L. McNaughton (1994). "Reactivation of hippocampal ensemble memories during sleep." Science 265(5172): 676–679.

Yang, S. R., H. Sun, Z. L. Huang, M. H. Yao and W. M. Qu (2012). "Repeated sleep restriction in adolescent rats altered sleep patterns and impaired spatial learning/memory ability." Sleep 35(6): 849–859.

Yu, J. Y., D. F. Liu, A. Loback, I. Grossrubatscher and L. M. Frank (2018). "Specific hippocampal representations are linked to generalized cortical representations in memory." Nat Commun 9(1): 2209.

Zhang, L. B., J. Zhang, M. J. Sun, H. Chen, J. Yan, F. L. Luo, Z. X. Yao, Y. M. Wu and B. Hu (2020). "Neuronal Activity in the Cerebellum During the Sleep-Wakefulness Transition in Mice." Neurosci Bull 36(8): 919– 931.
